# Adaptive Sampling-Based Information Collection for Wireless Body Area Networks

**DOI:** 10.3390/s16091385

**Published:** 2016-08-31

**Authors:** Xiaobin Xu, Fang Zhao, Wendong Wang, Hui Tian

**Affiliations:** 1 School of Software Engineering, Beijing University of Posts and Telecommunications, Beijing 10087, China; zfsse@bupt.edu.cn; 2 State Key Laboratory of Networking and Switch Technology, Beijing University of Posts and Telecommunications, Beijing 10087, China; wdwang@bupt.edu.cn (W.W.); tianhui@bupt.edu.cn (H.T.)

**Keywords:** wireless body area networks, data collection, information quantity, data sampling

## Abstract

To collect important health information, WBAN applications typically sense data at a high frequency. However, limited by the quality of wireless link, the uploading of sensed data has an upper frequency. To reduce upload frequency, most of the existing WBAN data collection approaches collect data with a tolerable error. These approaches can guarantee precision of the collected data, but they are not able to ensure that the upload frequency is within the upper frequency. Some traditional sampling based approaches can control upload frequency directly, however, they usually have a high loss of information. Since the core task of WBAN applications is to collect health information, this paper aims to collect optimized information under the limitation of upload frequency. The importance of sensed data is defined according to information theory for the first time. Information-aware adaptive sampling is proposed to collect uniformly distributed data. Then we propose Adaptive Sampling-based Information Collection (ASIC) which consists of two algorithms. An adaptive sampling probability algorithm is proposed to compute sampling probabilities of different sensed values. A multiple uniform sampling algorithm provides uniform samplings for values in different intervals. Experiments based on a real dataset show that the proposed approach has higher performance in terms of data coverage and information quantity. The parameter analysis shows the optimized parameter settings and the discussion shows the underlying reason of high performance in the proposed approach.

## 1. Introduction

In recent years, advances in microcircuits and medical sensing have made it possible to deploy battery-powered miniaturized sensors on, in, or around the human body for long-term healthcare monitoring [1,2,3]. These body sensors report their sensory data to a data sink via wireless communication channels. The data sink is a pre-defined portable device, such as a PDA or a cell phone worn on the human body. It may be linked to a remote healthcare agency through the cellular network and/or the Internet, for example. The body sensors and the sink together constitute a small-scale wireless sensor network (WSN), called wireless body area network (WBAN). Most types of health data change rapidly, especially electroencephalogram (EEG), electrocardiogram (ECG), etc. [1]. These data contain important information about the human body. In the traditional healthcare monitoring, health data are typically sampled at a very high frequency (for example, 100 Hz) to avoid loss of healthy information. A high data sampling frequency can be achieved easily in the traditional healthcare monitoring because most types of monitoring equipment are powered by bulk power system and they upload the sampled data via reliable cable transmission, whereas WBANs will face two challenges to achieve high sampling frequency. Firstly, since the WBAN sensors are battery-powered, high sampling frequency will shorten lifetimes of WBAN nodes [4,5,6]. Secondly, since in WBANs data are collected via wireless transmissions, the capability of transmissions will limit the data collection capability of WBAN as well [7]. To extend the lifetime of WBANs, various types of energy-efficiency approaches are proposed. Some of them try to design the network to minimize energy consumption [8,9,10,11]. Some of them collect a portion of sensed data and reconstruct the complete dataset at the cost of a tolerable error [12,13,14,15,16,17,18]. Limited by wireless communication capability, nodes have bounded transmission frequencies. Nevertheless, most of the existing data collection approaches cannot control the transmission frequency directly. Instead, they reduce transmissions by setting error bounds. In real WBAN applications, the most common scenario is that sensors sense data at a high frequency (for example, 100 Hz), but nodes can only upload data in a low frequency (for example, 1 Hz). Then nodes have to sample a portion of sensed values at a certain probability and upload these values to the data sink periodically. One of the most important questions in data collection is how to obtain information in an optimal way under the constraint that the sampling probability is limited. To the best of our knowledge, this is still an open question in data collection.

### 1.1. Motivation

**Figure 1:**
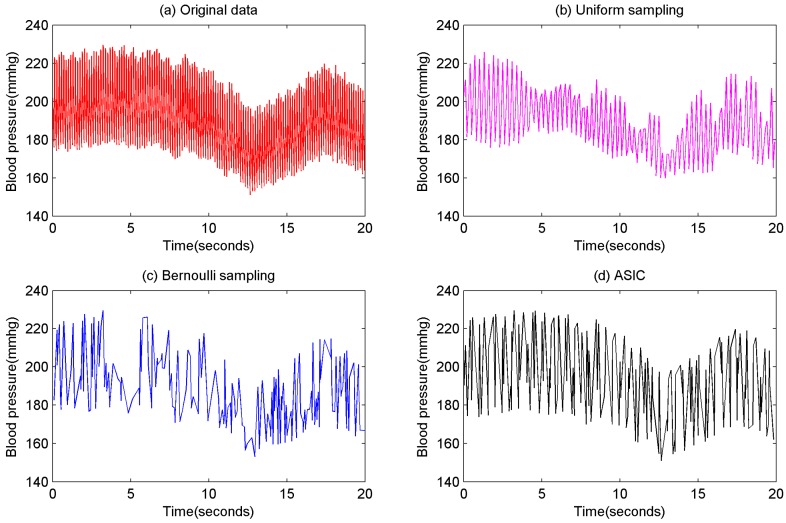
Results of sampling approaches, (**a**) original data; (**b**) uniform sampling; (**c**) Bernoulli sampling; (**d**) ASIC.

Although the core task of WBAN applications is to collect health information, most of the existing data collection approaches in WBAN focus on sensed data instead of information. These approaches collect data at the constraint of error bound [19,20,21,22,23,24,25,26], but they cannot control the sampling frequency directly and are not sensitive on information. Some sampling approaches [27,28,29,30,31] have been proposed to sample data from original sensed data at preset sampling probabilities. The uniform sampling approach [27] samples every value in the same time cycle, while the Bernoulli sampling [29] approach samples every value at the same probability. An example of these approaches is shown in Figure 1. This example uses the real blood pressure recordings from Dahl SS’ rats on a High Salt Diet [32]. The sampling probabilities of these approaches are both 0.1. It is obvious that some important information are lost through these approaches because they are not sensitive on information. There is a subtle difference between data and information. Data are the facts or details from which information is derived. So the sensors of WBAN applications should collect data from which users can extract optimized information. That motivated us to propose an information collection approach for WBAN.

### 1.2. Main Contributions

Information theory has already described the quantization of information: the smaller the data probability, the larger its information quantity. Accordingly, no matter what the scenario is, unusual data of the human body is always important because it has high information quantity. The ideal sampling result is the smaller data probability, the larger sampling probability so as to ensure users obtain uniformly distributed data (i.e., optimized information). According to this strategy, this paper builds a novel data collection approach named Adaptive Sampling-based Information Collection (ASIC). In the prior example, if ASIC is used to collect data at the same probability 0.1, the result is shown in Figure 1d. It is clearly shown that ASIC can collect most of important information from original data. The main contributions of this paper are as follows:

- A general form of sampling models is given. Based on this model, we define an information collection model. This model attempts to obtain uniformly distributed data which is considered to have optimized information. In this model, the information quantity of every single value in the original dataset is quantified and the sample probability for every single value is computed. The bigger the information quantity, the larger the sampling probability.
- Adaptive sampling-based information collection is proposed based on the information collection model. Adaptive sampling-based information collection is constituted by Adaptive Sampling Probability (ASP) algorithms and Multiple Uniform Sampling (MUS) algorithms. Adaptive sampling probability algorithms are used to compute and update sampling probabilities of different values. Multiple uniform sampling algorithms are used to determine which values should be sampled. These algorithms are performed by body nodes periodically.
- A series of simulations are conducted to evaluate the performance of adaptive sampling- based information collection on the basis of a real dataset. Experimental results show that ASIC has higher performance in terms of data coverage and information quantity. Finally, we discuss the effects of various parameters and the underlying reason why ASIC has high performance.

The remainder of the paper is organized as follows: Section 2 discusses related works. Section 3 shows the system model, the data sampling model and the information collection model. Section 4 proposes an adaptive sampling-based information collection which is constituted by the adaptive sampling probability algorithm and the multiple uniform sampling algorithm. Section 5 shows experimental results. Section 6 concludes the paper.

## 2. Related Works

Because of its battery-powered sensor nodes, the energy efficiency of a WBAN becomes one of the critical problems in real-world WBAN applications. To achieve energy efficiency, there are many researches in the fields of network design and data collection. Network design proposes a network layer approach to achieve energy efficiency. Reference [8] introduces a new robust optimization model and a new algorithm for designing wireless body area networks taking into account healthy data generation uncertainty. The algorithm is based on a fast ant-inspired evolutionary algorithm for optimal network design presented in [9]. Elias et al. introduced flow optimization models for designing wireless body area networks [10,11].

To collect data efficiently, various types of data collection approaches are proposed for WBAN. A system which has a scalable platform that requires minimum human interaction during setup and monitoring is proposed in [12]. Reference [13] discusses the security and privacy protection of data collected from a WBAN and fine-grained distributed data access control for sensitive and private patient medical data. A real-time monitoring system is proposed in [14]. The main techniques developed are the caching for multi-source data stream support, complex range expression processing with interval skip lists, and historic data processing for sliding window condition monitoring support. The condition monitoring query language and efficient processing mechanisms to support the complex monitoring are presented as well. Ángel et al. proposed a model-driven approach based on a meta-model which has been designed to define and specify interaction with sensors [15]. They also introduced the coordinator, i.e., component-based software for handling sensor models and improving the integration of new sensors. Han et al. proposed the Multi-valued and Ambiguous Scheme to capture data confidentiality in the Cloud-assisted Wireless Body Area Networks [16]. The approach of combining the scheme with existing encryption schemes provides a general paradigm for deploying applications. Reference [17] proposes a scheme that detects selfish behavior and prevents its occurrence in mobile ad hoc networks. The proposed scheme uses an adaptive threshold algorithm to detect selfish behavior, and prevents it through a repeated games scheme. A scalable storage and processing infrastructure are proposed for large scale WBANs system in [18]. This infrastructure is shown to be able to efficiently handle the large size of data generated by the WBANs system, by storing these data and performing analysis operations on it.

To reduce transmissions, various approaches are proposed. BaySail [19] is proposed which incorporates the knowledge of the suppression scheme and application-level redundancy in Bayesian inference. Our scheme is specifically designed for BSNs, where nodes are likely fully connected and overhearing among sensor nodes is possible. Wu et al. proposed a scheme to achieve data compression by temporal and spatial correlations [20]. They presented algorithms to determine the partial ordering and discussed the design of the underlying MAC protocol to support the compression model. Reference [21] proposes to use the block sparse Bayesian learning framework to compress/reconstruct nonsparse raw FECG recordings. This framework can greatly reduce code execution in CPU in the data compression stage. Radović et al. proposed a sparsity model that allows the use of Compressive Sensing (CS) for the online recovery of large data sets, exploiting Principal Component Analysis (PCA) to capture the spatial and temporal characteristics of real signals [22]. They also proposed an extensive validation of the framework used in conjunction with CS as well as with standard interpolation techniques, testing its performance for real world signals. Reference [23] proposes approaches to increase the reliability of WBANs, they minimize the power consumption and sampling-rate in the Multipath Fading Channels (MFCs) between BWSs and GW. Hung et al. presented a benchmark that offers a comprehensive empirical study on the performance comparison of the model-based compression techniques in sensor networks [24]. They re-implemented several state-of-the-art methods in a comparable manner, and measured various performance factors with our benchmarks, including compression ratio, computation time, model maintenance cost, approximation quality, and robustness to noisy data. In [25], real-time collection of the sensed data and real-time processing of these data series are reviewed in sensor networks. To solve the new optimization problems in a Blind Compressed Sensing (BCS) framework, Majumdar et al. derived algorithms based on the Split Bregman approach [26]. The resulting recovery results are considerably better than those of previous techniques, in terms of the quantitative and qualitative evaluations. All these approaches can reduce data transmissions by setting the error bound. However, they cannot control upload frequency of body sensors directly. In the most common scenario of WBAN, the upload frequency is limited by the wireless communication capability [7]. It is the weakness of prior approaches.

To reduce transmissions, some sampling approaches are proposed for WSNs and WBANs as well. Most traditional approaches use uniform sampling methods to collect data [27]. Through uniform sampling, every value is sampled in the same time interval. A spatial uniform sampling approach is proposed in WSNs [28], through spatial uniform sampling, same numbers of sensors upload their sensed data at same time interval. A Bernoulli sampling approach is proposed in [29], through Bernoulli sampling, every sensed value has the same probability to be uploaded to the sink. These two sampling approaches can control sampling probability (i.e., upload frequency) directly. However, sampling probabilities in these approaches are computed on the basis of accuracy requirements. In other words, in these approaches, sampling probabilities are determined by the accuracy requirement as well. As shown in Figure 1, these approaches have a high loss of important information. An adaptive sampling based approach is proposed in [30], and a compressed sensing based sampling approach is proposed in [31]. Nonetheless, these approaches cannot control upload frequency directly either. In this paper, we propose an approach which is suitable in real WBAN applications. This approach can directly control sampling probability. While the prerequisite of a certain sampling probability, body sensors sample and upload data which contains optimized information via our approach.

## 3. Modeling

In this section, we first describe the system model of data collection in WBAN. Then we define sampling models and give their general form. After that, we define an information collection model to obtain optimized information.

### 3.1. System Model

In the physical world, health data change continuously but body nodes can only sense values periodically. To describe the system model, we define health data and original sensed data at first.

**Definition 1.**  Health data. Health data can be denoted as a dataset of two-tuples, HD={(v,t)|v=f(t)),t_start≤t≤t_end}, where t denotes time, v denotes value, t_start and t_end denote start and end of time interval, respectively.

**Definition 2.**  Original sensed data. The sensing frequency of body nodes has an upper limit limited by hardware. That means a node can only sense a portion of the health data and the amount of data sensed by a node has an upper limit. If $T=\{t_{1},t_{2},\ldots ,t_{n}\}$ denotes the times when a body node senses data. Original sensed data can be denoted as a discrete dataset of two-tuples: OD={(v,t)|v=f(t),t∈T} , which is a subset of HD. Existing approaches usually collect data at a certain frequency $f_{s}$, for example, 100 Hz, then T={Δt,2Δt,…,nΔt}, where Δt is the time interval of sensing, $\Delta t=\frac{1}{f_{s}}$.

**Figure 2:**
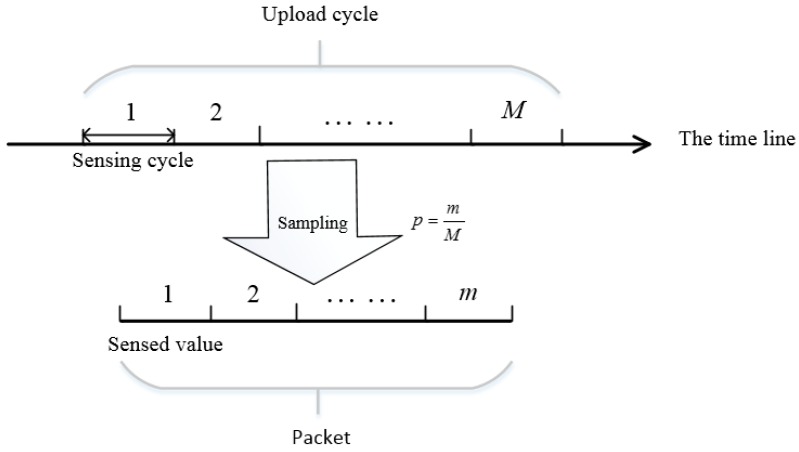
A typical scenario of data sampling in WBAN.

Limited by the network stack, a body sensor has to upload its data at a low frequency $f_{u}$ to guarantee that packets can be successfully uploaded, for example, 1 Hz. Then every time a body sensor uploads its sensed data, there are *M* sensed values to be uploaded, $M=\frac{f_{s}}{f_{u}}$. However, the packet provided by network stack has a size limit, in other words, one packet can only carry several sensed values. We denote the number of values one packet can carry as *m*. Then, the body sensor has to sample and upload *m* values from *M* sensed values. We denote the sampling probability as *p*, $p=\frac{m}{M}$. The details are shown in Figure 2.

### 3.2. Data Sampling Model

To describe data sampling models, we give the definition of sampled data first.

**Definition 3.**  Sampled data. Since the energy of one body sensor is limited, one sensor has to sample a portion of the original sensed data and upload it to the sink. The uploaded data is called sampled data. Sampled data can be denoted as a dataset SD, which is a subset of OD. The general form of data sampling approaches can be denoted as: SD = data_sampling(OD).

The uniform sampling approach samples every value in the same time interval. If we denote the times when the body sensor samples data as $ST=\{st_{1},st_{2},\ldots ,st_{i},\ldots \}$, through uniform sampling, the following equation is satisfied: $st_{2}-st_{1}=st_{3}-st_{2}\ldots =st_{i}-st_{i-1}=\ldots =interval$. Where interval denotes the time interval of sampling.

The Bernoulli sampling approach samples every value with the same probability *p* at the same time. Through Bernoulli sampling, the following equation is satisfied: ∀v, $P_{s}(v)=p$. Where $P_{s}$ denotes the sampling probability function.

Neither of these approaches is sensitive to the information of sensed values. In this paper, we quantify the importance of sensed values and build a sampling model based on information theory for the first time. In information theory, data with smaller probability has larger information quantity. Accordingly, no matter what the scenario is, unusual body conditions data is always important to WBAN applications. Although rarer values have higher importance, common values still need to be sampled, but with a low probability. The ideal sampling result is uniformly distributed data. We define information-aware adaptive sampling as follows:

**Definition 4.**  Information-aware adaptive sampling. Information-aware adaptive sampling is to obtain optimized information at a preset sampling probability. If we denote the original values as $V=\{v_{1},v_{2},\ldots ,v_{M}\}$, denote the sampling probability as p, denote the sampled values as $SV=\{sv_{1},sv_{2},\ldots ,sv_{m}\}$, through this approach, the following equations is satisfied: (1) m=pM; (2) SV is uniformly distributed. We will show the details of information-aware adaptive sampling in the rest of Section 3.

### 3.3. Information-Aware Adaptive Sampling Model

In information theory, self-information and entropy are measures of information quantity [33]. Named after Boltzmann’s H-theorem, Shannon denoted the entropy *H*(*X*) of a discrete random variable *X* with possible values $\left\{x_{1},x_{2},\ldots ,x_{n}\right\}$ and probability mass function *P(X)* as follows:

(1) $$I(X)=-\log_{b}(P(X))$$

(2) $$H(X)=E(I(X))=E(-\log_{b}(P(X)))$$

Here *I* is the self-information of a certain value *X* and *E* is the expected value operator. *I*(*X*) itself is a random variable, and *b* is the base of the logarithm used. Common values of *b* are 2, Euler’s number *e*, and 10, and the unit of entropy is the bit for *b* = 2, the nat for *b* = *e*, and the dit (or digit) for *b* = 10. When taken from a finite sample, the entropy can be written explicitly as:

(3) $$H(X)=\sum_{i=1}^{n}P(x_{i})I(x_{i})=-\sum_{i=1}^{n}P(x_{i})\log_{b}P(x_{i})$$

The measure of entropy should be maximal if all the outcomes are equally likely (uncertainty is highest when all possible events are equiprobable) [33]:

(4) $$H_{n}(p_{1},p_{2},\ldots ,p_{n})\leq H_{n}(\frac{1}{n},\frac{1}{n},\ldots ,\frac{1}{n})=\log_{b}n$$

Since the value with larger self-information is more important, we define the importance of a certain value *x_i_* as a monotonically increasing function of its self-information:

(5) $$Importance\left(x_{i}\right)=b^{I(x_{i})-\log_{b}n}=\frac{1}{np_{i}}$$

where *p_i_* denotes the probability of *x_i_*, *n* denotes the number of the random variable *X*’s possible values, then the mean probability of *X* is $\frac{1}{n}$. When *p_i_* is equal to $\frac{1}{n}$, the importance of *x_i_* is equal to 1. In the same way, when *p_i_* is larger or smaller than $\frac{1}{n}$, the importance of *x_i_* is smaller or larger than 1.

**Theorem 1.**  Suppose that there is a discrete random variable X with values $\left\{x_{1},x_{2},\ldots ,x_{n}\right\}$ and probabilities $\left\{p_{1},p_{2},\ldots ,p_{n}\right\}$, when sampling probability p satisfies that $\forall i,p\leq np_{i}$, information-aware adaptive sampling is achieved if and only if the sampling probability of every arbitrary value x_i_ is $p\times Importance\left(x_{i}\right)$.

**Proof:** 

- Sufficiency:
  We denote sampling probability of *x_i_* as $P_{s}\left(x_{i}\right)$.
  $∵\forall x_{i},P_{s}\left(x_{i}\right)=p\times Importance\left(x_{i}\right)=\frac{p}{np_{i}},P_{s}\left(x_{i}\right)\leq 1$, and the probability of *x_i_* is *p_i_*
  $∴\forall x_{i}$, the number of sampled *x_i_* to total number of values in original dataset ratio is $p_{i}\times P_{s}\left(x_{i}\right)=\frac{p}{n}$.
  So, $\forall x_{i}$, the number of sampled *x_i_* is constant, the distribution of sampled values is uniform. The total number of sampled values to total number of values in the original dataset ratio is equal to *p*.
  Information-aware adaptive sampling is achieved. Proof of sufficiency ends.
- Necessity: We denote the total number of values in original dataset as *m*, and the sampled number of *x_i_* as *m_i_*. If Information-aware adaptive sampling is achieved, $m_{1}=\ldots =m_{i}=\ldots =m_{n}$, and $\sum_{i=1}^{n}m_{i}=pm$. So, $\forall x_{i},m_{i}=\frac{pm}{n}$.
  $∵\forall x_{i},P_{s}\left(x_{i}\right)=\frac{m_{i}}{p_{i}m}$
  $∴\forall x_{i},P_{s}\left(x_{i}\right)=\frac{p}{np_{i}}=p\times Importance\left(x_{i}\right)$
  Proof of necessity ends.

In WBAN applications, the original sensed data is a set of continuous values and the number of elements in this set is limited. Since information-aware adaptive sampling can be achieved based on a discrete random variable, we can discretize the dataset and sample data in the same way. Equal width interval binning [34] is the most popular method for discretizing data. It involves sorting the observed values of a continuous feature and dividing their range into equally sized bins. We use this approach to discretize data. Since the minimum value and the maximum value are important to users, we divide these two values into two bins. If we denote the minimum value and the maximum value as *v_min_* and *v_max_*, denote the number of bins as *n_b_*, then the width of one bin can be computed as: $width=\frac{v_{max}-v_{min}}{n_{b}-2}$. The ranges of bins are $\left\{\{v_{min}\},(v_{min},v_{min}+width]\ldots (v_{max}-width,v_{max}),\{v_{max}\}\right\}$. According to Equation (5), if the probability of sensed data in an arbitrary bin *b_i_* is *p_i_*, the importance of data in this bin is:

(6) $$Importance\left(b_{i}\right)=\frac{1}{n_{b}p_{i}}$$

According to Theorem 1, to achieve information-aware adaptive sampling, the sampling probability of sensed data in this bin is:

(7) $$P_{s}\left(b_{i}\right)=\frac{p}{n_{b}p_{i}}$$

## 4. Adaptive Sampling Based Information Collection

In practical WBAN applications, body sensors sense values at a high probability but upload values at a low frequency. Then we propose an adaptive sampling-based information collection approach which is performed by body sensors periodically. This approach is constituted by Adaptive Sampling probability (ASP) algorithms and Multiple Uniform Sampling (MUS) algorithms. We describe the parameters used in our approach at first, and then show the details of these algorithms.

### 4.1. Information Collection in WBAN

To describe the adaptive sampling-based information collection approach clearly, we show the major parameters of our approach in Table 1.

In real WBAN applications, adaptive sampling-based information collection is performed in three steps:

- Parameter setting: Users determine *f_s_*, *f_u_*, *m* and *n_b_*. The sink computes the parameter *p*.
- Initialization: The sink broadcasts parameters to all the body sensors. Body sensors begin to sense healthy data.
- Data sampling: Every sensor performs the adaptive sampling probability algorithm and the multiple uniform sampling algorithm periodically to achieve information-aware adaptive sampling at every upload cycle. Through the adaptive sampling probability algorithm, upload probabilities of different sensed values are computed and updated in every cycle. Through the multiple uniform sampling algorithm, nodes sample and upload sensed values to the sink. The details of these two algorithms are shown in Section 4.2 and Section 4.3.

### 4.2. Adaptive Sampling Probability Algorithm

The adaptive sampling probability algorithm is proposed to compute and update the ranges of bins and sampling probabilities of different sensed values when WBAN nodes are sensing data. In this algorithm, Equation (7) is used. In Equation (7), sampling probability p and the number of bins nb are computed in the parameter setting step. Then the parameter P which denotes probabilities of sensed values in different bins should be computed by body sensors. The details of adaptive sampling probability algorithm are as follows. Firstly, ranges of bins and sampling probabilities are computed. Secondly, an array of counters is set to count the number of values in different bins. Thirdly, the ranges of bins and sampling probabilities are computed and updated through Equation (7). The details are shown in Algorithm 1.

**Algorithm 1:** Adaptive Sampling Probability algorithm  **Input:**
*n_b_*, *p*, $V=\{v_{1},v_{2},\ldots v_{M}\}$  **Output:** boundaries <$v_{min}$, width, $v_{max}$>, sampling probabilities *P_s_* 1   Compute boundaries: *v_min_* = *v*_1_; *v_max_* = *v*_1_; 2   **for** (*i* = 1; *i* < *M* + 1; *i*++) { 3   **if** (*v_min_* > *v_i_*) *v_min_* = *v_i_*;     4  **if** (*v_max_* < *v_i_*) *v_max_* = *v_i_*;} 5   Compute numbers of values in different bins: 6   numbers={0,0,…,0}//the length is *n_b_*. 7   *index = 0;*
$width=\frac{v_{max}-v_{min}}{n_{b}-2}$*;* 8   **for** (*i* = 1; *i* < *M* + 1; *i*++) { 9   **if** (*v_min_* == *v_i_*) *index* = 1; 10   **else if** (*v_max_* == *v_i_*) *index* = *n_b_*; 11   **else**
$index=\left\lceil \frac{v_{i}-v_{min}}{width}\right\rceil +1;$ 12   $numbers_{index}++;$} 13   Compute sampling probabilities: $r=\frac{pM}{n_{b}}$ 14   **for**  (*i* = 1; *i* < *n_b_* + 1; *i*++) $P_{s}(i)=\frac{r}{numbers_{i}}$.

Through the adaptive sampling probability algorithm, sampling probabilities of sensed values in different bins are computed. According to these probabilities, body sensors will sample values and upload them to the sink.

### 4.3. Multiple Uniform Sampling Algorithm

Since every node maintains ranges of bins and sampling probabilities of values in these bins, then sampling probability of every sensed value can be computed according to these parameters. For sensed values in the same bin, the sampling probabilities are the same. Then we can perform uniform sampling or Bernoulli sampling in the same bin. In this paper, we perform uniform sampling in the same bin, this algorithm is called multiple uniform sampling algorithm. In this algorithm, every node maintains an array to record the time intervals between sensed values and latest sampled values in the same bins. The expected sampling intervals of values in different bins can be computed as the reciprocal of the sampling probabilities of values in these bins. When the time interval of values in a certain bin is equal to or larger than the expected sampling interval, the node uploads the sensed value to the sink. We initialize the time intervals to be the same as the expected sampling intervals to ensure that the first value of every bin can be uploaded. This strategy can also ensure that the minimum and the maximum values are sampled. The details of the multiple uniform sampling algorithm are shown in Algorithm 2.

**Algorithm 2:** Multiple Uniform Sampling algorithm  **Input:** <$v_{min}$, width, $v_{max}$>, *P_s_*, $V=\{v_{1},v_{2},\ldots v_{M}\}$  **Output:** Sampled values 1   Counter initialization: $counter=\{\frac{1}{P_{s}(1)},\ldots ,\frac{1}{P_{s}(n_{b})}\}$; 2   Sampling: 3   **for** (*i* = 1; *i* < *M* + 1; *i*++) { 4   **if** (*v_min_* == *v_i_*) *index* = 1;     5   **elseif** (*v_max_* == *v_i_*) *index* = *n_b_*;     6   **else**
$index=\left\lceil \frac{v_{i}-v_{min}}{width}\right\rceil +1;$ 7   **if** ($counter_{index}\geq \frac{1}{P_{s}(index)}$)  { 8   Sample $v_{i}$; $counter_{index}-=\frac{1}{P_{s}(index)}$;} 9   $counter_{index}++$;}

Through the adaptive sampling probability algorithm and the multiple uniform sampling algorithm, the sampling result of every sensor can achieve approximately uniform.

## 5. Performance Evaluation

To evaluate the performance of our approach, we introduce the real dataset of blood pressure recordings from Dahl SS rats on High Salt Diet [32] and design a series of experiments to verify performance of our approach in terms of data coverage and entropy of sampled data. After that, we analyze the effects of parameter setting and discuss our approach.

### 5.1. Experimental Setup

In the real dataset, the record frequency is 100 Hz, which means that *f_s_* = 100 Hz. We set the parameter *m* to be 10. In other words, when sensor nodes upload sensed data to the sink, one packet can carry 10 sensed values. These experiments are simulated on the basis of 90,000 values from nine rats. The upload frequency and the number of bins vary in our simulations. We use ASIC to express the adaptive sampling-based information collection approach in this paper. We use U sampling to express the uniform sampling based approach [27]. We use B sampling to express the Bernoulli sampling based approach proposed in [29]. We compare ASIC with U sampling and B sampling in terms of data coverage and entropy.

### 5.2. Data Coverage

To collect information of sensed data comprehensively, the sampled data should cover as large range of sensed values as possible. Moreover, data in different ranges should be sampled. To evaluate the data coverage, we investigate the data range and coverage of sampled data.

● Data range

**Figure 3:**
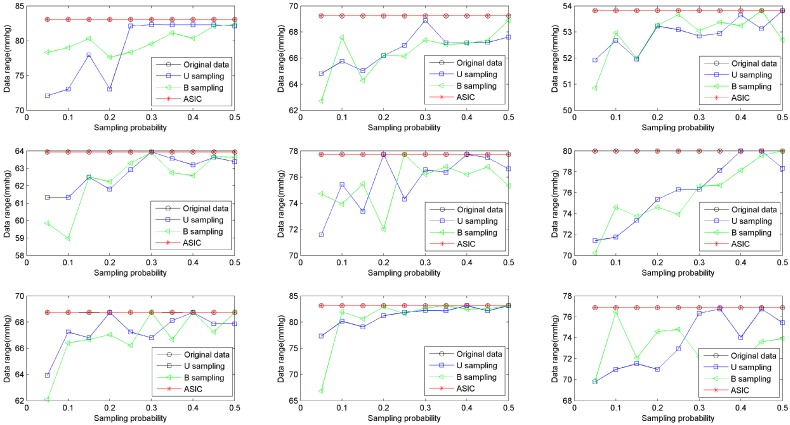
Data range affected by sampling probability in a real dataset.

Data Range (DR) is used to evaluate the range of data. DR is computed as: *DR* = *v_max_* − *v_min_*. To compare the data range of ASIC with that of B sampling and U sampling, we build simulations on the basis of sensed values from all nine rats. The upload frequency increases from 0.5 Hz to 5 Hz. Then the sampling probability increases from 0.05 to 0.5. The number of bins is set to be 5, i.e., *n_b_* = 5. Ranges of data collected from datasets of nine rats are shown in Figure 3. The figure shows that ASIC can ensure that the range of collected data is the same as the range of the original sensed value. In other words, the maximum and the minimum values of healthy data are always sampled via ASIC. In most WBAN applications, the maximum and the minimum values of healthy data are very important for healthcare monitoring. This result verifies that ASIC can always capture these important records.

● Coverage of sampled data

**Figure 4:**
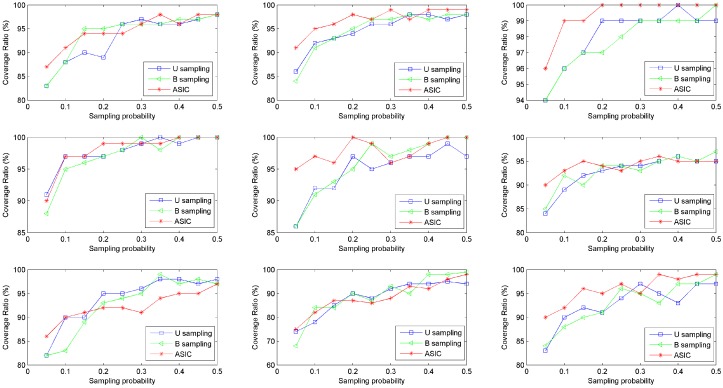
Coverage of sampled data.

To investigate the coverage of sampled data, we divide the range of sensed values into 100 subranges with uniform width at first. For any subrange, if there are at least one value in this subrange sampled by data sampling approach, we call that this subrange is covered. We use Coverage Ratio (CR) to evaluate the coverage of these subranges. The computation is $CR=\frac{number\_of\_covered\_ranges}{number\_of\_total\_ranges}\times 100\%$. The parameter settings are the same as the prior simulations. Coverage ratios of data collected from nine rats are shown in Figure 4. It is shown that ASIC has higher coverage ratio especially when the sampling probability is low. In other words, values in most subranges will be sampled via ASIC. The probability of losing important information will be smaller if WBAN collects data via ASIC.

### 5.3. Information Quantity

**Figure 5:**
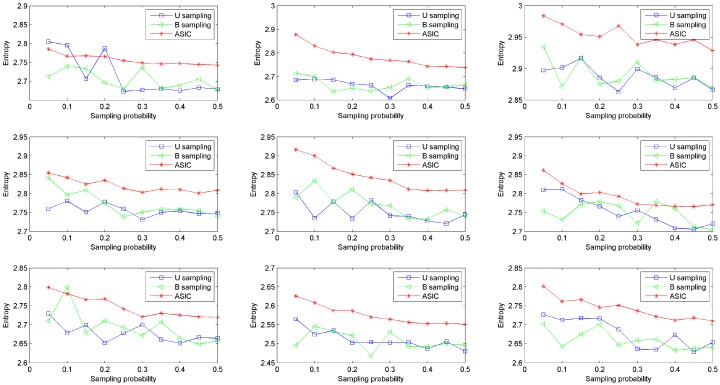
Comparison of entropy.

To measure information quantity of sampled data, we introduce entropy. Traditional entropy is computed on the basis of discrete variable. The computation is shown in Section 3.3. To evaluate the information quantity of a dataset with continuous values, we first divide the range of the dataset into eight intervals with uniform width. After that, we discretize the dataset into a discrete variable with eight possible values. Then we can compute the entropy of this dataset according to Equation (3). The base of the logarithm used is set to be 2, i.e., *b* = 2. The other parameter settings are the same as the prior simulations as well. Information quantities of data collected from nine rats are shown in Figure 5. This figure shows that data sampled via ASIC has higher entropy than data sampled via U sampling and data sampled via B sampling in most conditions. The entropy of data sampled via ASIC decreases smoothly as sampling probability increases. While the entropies of data sampled via the other two approaches fluctuate as sampling probability increases. In other words, ASIC can acquire more information stably than the other two sampling approaches. In line with this result, in real WBAN applications, using ASIC can collect healthy data in an optimal and stable way, i.e., sampled data has high information quantity.

### 5.4. Analysis of Parameter Setting

**Figure 6:**
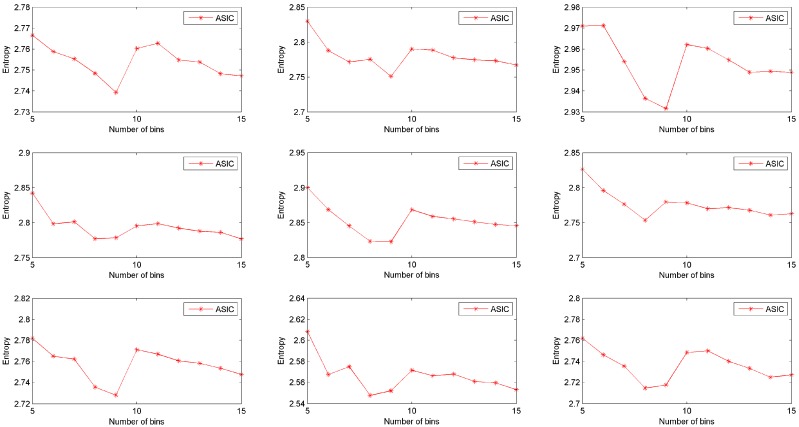
Effects of number of bins.

To investigate the impact of parameters in our approach, a group of experiments is simulated based on the real dataset. In these experiments, the sampling probability is set to be 0.1, while the number of bins varies from 5 to 10. Information quantities of data collected from nine rats are shown in Figure 6. In this figure, data collected by ASIC has higher entropy when the number of bins equals to 5 or 10. In other words, when the number of bins is set to be 5 or 10, body sensors can collect more information via ASIC. When the number of bins is larger than 10, the entropy of sampled data decreases as the number of bins increases, i.e., ASIC will collect less information when the number of bins is larger. This result demonstrates that the number of bins should not be too large. When ASIC discretizes the original dataset, it is not necessary to divide the range of the dataset into too many bins (5 or 10 is a good quality selection).

### 5.5. Discussion

To discuss the underlying reason why ASIC has a higher performance in data collection, we investigate the distribution of values collected through ASIC by performing two groups of experiments. These experiments are built on the basis of a real and a synthetic dataset. The parameter settings of these experiments are the same. The number of bins is set to be 5, the sampling probability is set to be 0.1. The other parameter settings are the same as the prior experiments.

**Figure 7:**
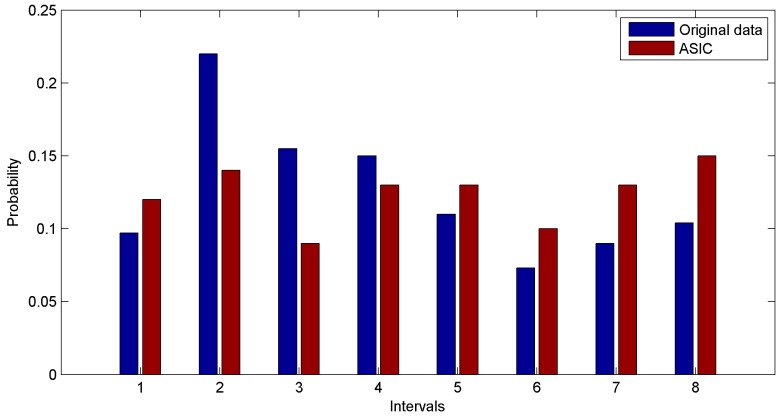
Comparison of distributions of data collected from real dataset.

The first group of experiments is performed based on the real dataset. We build our simulation on the basis of 1000 values sensed by the first rat. We discretize the range of real dataset into eight intervals and plot the probability distribution function of datasets. Probability distribution functions of the original dataset and the data sampled through ASIC are shown in Figure 7. In Figure 7, the data sampled through ASIC approximately follows a uniform distribution. In other words, the numbers of values in different intervals are almost the same. Thus the important data and the information of original dataset are hard to lose. This is the real reason why data sampled via ASIC has high coverage ratio and high entropy.

**Figure 8:**
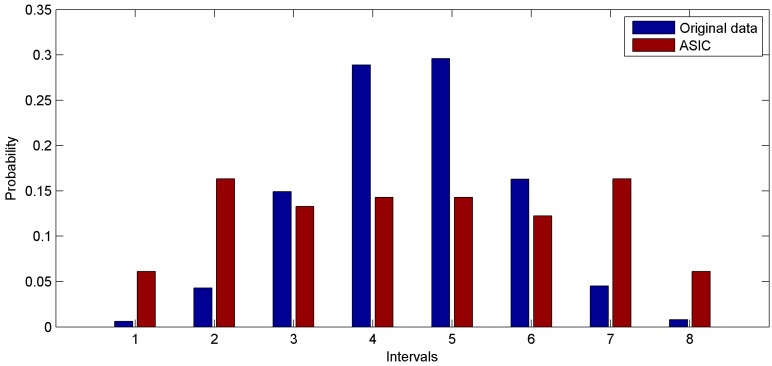
Comparison of distributions of data collected from synthetic dataset.

To discuss the distribution of values collected via ASIC in the common condition, the second group of experiments is performed on the basis of a synthetic dataset. The synthetic dataset follows normal distribution: *N* (200, 7^2^). The parameter settings are the same as in the first group of experiments. The probability distribution functions of the original data and the data collected via ASIC are shown in Figure 8. Similar with Figure 7, data sampled through ASIC approximately follows a uniform distribution as well. This result demonstrated that in the common condition, ASIC can acquire uniformly distributed data from the original dataset.

Through ASIC, the probabilities of collected values in different intervals are approximately equal, so values with lower probability are sampled at a higher probability. Then ASIC can capture important information at a high probability because values with lower probability have more information according to information theory. In summary, the good performance of ASIC is determined by the strategy used in the Information-aware Adaptive Sampling Model, which is, to collect uniformly distributed data.

## 6. Conclusions

Data collection of WBAN aims to sample data from the human body. Existing approaches mainly control the sampling probability by setting the error bound. However, they cannot control the sampling probability directly. The practicability of these approaches is limited by the capability of wireless transmission. In this paper, we put forward a novel approach to collect optimized information at a preset sampling probability. This approach intends to collect uniformly distributed data which is considered to have optimized information. Information-aware adaptive sampling is proposed in this paper to provide a general approach to obtain uniformly distributed data. Adaptive sampling-based information collection is put forward for WBAN to achieve optimized data collection. Experimental results show that our approach has higher performance in terms of data coverage and information quantity.

## Figures and Tables

**Table 1:** Parameters Used in ASIC.

Parameter	Meaning
*f_s_*	Sensing frequency of body sensors.
*f_u_*	Uploading frequency of body sensors.
*M*	Total number of values sensed by one body sensor in one upload cycle, *M = f_s_*/*f_u_*.
*m*	Number of sensed values one packet can carry.
*p*	Overall sampling probability, *p* = *m*/ *M*.
*n_b_*	Number of bins in data discretization.
*P*	Set of probabilities of values in different bins, computed and updated through ASP in every body sensor.
*P_s_*	Sampling probabilities of values in different bins, computed and updated through ASP in every body sensor.
